# Possibly mad? Marital murder in the early twentieth century: a matched-case gender analysis of forensic psychiatric investigations in Sweden

**DOI:** 10.1177/0957154X231191691

**Published:** 2023-09-27

**Authors:** Malin Hildebrand Karlén, Thomas Nilsson

**Affiliations:** University of Gothenburg, Sweden

**Keywords:** Forensic psychiatric investigations, gender, matched case study, partner homicide, Sweden, 1930s

## Abstract

This study illustrates the impact on forensic psychiatric investigations (FPI) of time-specific scientific theories and moral normative considerations. A comparative historical perspective illustrates historical FPI procedures (i.e. methodology and focus), based on two matched FPI case reports from the 1930s: a man and a woman who had shot their respective spouses. First, in the analysis, a comparison was made between the two cases regarding assessment procedure and focus, applying a gender perspective, and second, stability and change in FPI praxis between the 1930s and the 2020s were identified. Similarities and differences were discussed based on changes in FPI praxis and influence of explanatory models within psychiatry. This can aid understanding of historical bias and indicate current bias and its risks to FPI reliability.

## Introduction

Since the eighteenth century, various types of forensic psychiatric investigations (FPI) have been conducted in Sweden and, since the beginning of the twentieth century, in a similar manner to today (Bennet, 2020: 89–91; for background, see Lidberg, 2004). The FPI reports have extensive consequences for the individual – recommending forensic psychiatric care or prison – and they affect society’s perception of criminal sanction. FPIs take place at the interplay between law and psychiatry and demarcate what kind and degree of mental conditions should be considered sufficient for an alternative criminal sanction due to mental incapacity. Despite the intended objectivity of FPIs, they are carried out in a social context, mirroring norms and values relating to the contemporary psychiatric praxis, as well as general attitudes in society. For example, a study has shown that women and men may today are assessed somewhat differently within FPIs (Sygel et al., 2017). Also, in the 2000s, women who underwent an FPI were considered not guilty due to severe mental disorder (SMD) to a greater extent than men (Trägårdh, Nilsson, Granath and Sturup, 2016). This kind of systematic difference relating to gender could be a potential threat to the rule of law if men and women are not assessed equally (see Sygel et al., 2017), but it is difficult to observe such contemporary differences since the assessor always risks being unaware of or underestimating the impact of current norms (including gender) (Berg, 2018: ch. 6; Johannisson, 2015; Qvarsell, 1982: chs 8, 12). Historical FPIs offer the chance to identify how contemporary scientific knowledge and how time-specific values and norms affected FPI recommendations in the early twentieth century. These insights can also aid in discerning areas where contemporary FPIs are vulnerable to being influenced by values and norms that should not affect assessment praxis, such as factors related to gender.

## Forensic psychiatric investigations and Swedish law

In Sweden during the twentieth century, FPIs were carried out after referral from the court, in approximately the same manner as today. The purpose of these FPIs was to examine whether the accused person fulfilled medico-legal requirements for exemption from a legal penalty and instead was recommended for forensic psychiatric care. According to the Swedish penal law from the 1930s (Penal Code 5 chapt §5), an exemption from a legal penalty existed when an offender was ‘mentally ill’, or had ‘come into such a state due to no fault of their own, that they are out of their mind,^1^ should not be punished for acts committed within this state’. Acts committed by a person with mental incapacity due to a developmental disorder, dementia, etc. [*sinnesslö*] should also be considered exempt from punishment. If the person did not conform to the criteria of §5, but his or her mental state still deviated from the normal (or whose ‘spiritual capacities’^2^ had been disturbed without any fault of their own), then the court could grant a reduced sentence depending on the circumstances of the case (Penal Code 5 chapt §6).

The Swedish law has changed since the 1930s and concepts have been adapted to contemporary scientific knowledge. One rather substantial change was the introduction of the medico-legal concept of SMD. This term is central to FPIs, and has been elaborated during the present century. As the legislative history shows, this concept maintains historical focus on the exemption from a prison sentence of those with psychotic-like states and severe forms of intellectual disability. However, more diffuse states have also been relevant to psychiatric decision-making regarding exemption due to mental incapacity, although they are more difficult to evaluate based on clear legal and psychiatric diagnostic guidelines alone. In such cases, the forensic psychiatrist’s reasoning regarding what should constitute loss of reality orientation is more likely to be affected by the expert’s own ideas and moral values (exemplified in choice of information to underpin conclusions, theoretical framework chosen to explain the person’s psychological state and its effect on the criminal behaviour). To illustrate this, two matched cases have been explored in the present study: a man and a woman, each undergoing an FPI for having murdered their respective spouses.

## Aim

The overall aim of this study was to gain a better understanding of how ‘soft factors’ within forensic psychiatric decision-making could impact FPI praxis, using two cases from around the 1930s. By analysing these cases, it was possible to illustrate how contemporary scientific and social context could influence FPIs, including how the application of concepts and focus in the reports appears to be time-dependent due to specific conceptualizations of factors such as gender. This can not only aid the understanding of historically relevant bias, but also, due to a sharper contrast, indicate current forms of bias and their associated risks in today’s FPI praxis (see Dror and Murrie, 2018). The study also contributes with new perspectives on how today’s FPI praxis can be vulnerable to bias by illustrating how praxis fluctuates historically: *how* FPIs are conducted (i.e. methodology) and *what* is focused on (i.e. information considered central to the FPI conclusion).

## Method

A comparative historical perspective was used to illustrate historical FPI assessment procedures, using two written FPI case reports from around the 1930s. The methodology and specific issues were examined in two ways: (a) comparing two cases regarding assessment procedure and focus, as well as applying a gender-perspective on this praxis, and (b) comparing aspects of FPI praxis in the 1930s with that in the 2020s (based on established guidelines for FPIs^3^ [HSLF-FS 2015:31] and the authors’ extensive experience of FPI praxis), and discussing its historical stability and change.

### Selection of cases: W and M

To understand more about how contemporary norms and stereotypes relating to gender can impact the FPI process, a historical gender-matched case study was conducted. An in-depth analysis was made of two FPIs, those of a man (M) and a woman (W) who had shot and killed their respective spouses. Women commit fewer crimes than men, both now and historically, leaving men in a considerable majority within historical FPI records. According to our investigations, women who had committed homicide (adult victim) were very uncommon, and almost all would have been sent for an FPI, as is still the case today (see: Lidberg and Wiklund, 2004; Trägårdh et al., 2016). The study was initiated when one of the authors (TN) had identified an FPI from the early 1930s with a female perpetrator. Since this was a rare occurrence, criteria were created from this female case to find a male match: (1) having murdered his spouse, (2) during intoxication, and (3) the FPI having been conducted around the start of 1930s. All cases archived between 1928 and 1935 at one of the two FPI units in Sweden were screened, based on these criteria, and only one identified male case matched these criteria: the case of M who underwent an FPI in the late 1920s. Both the FPI reports and the court’s sentences, including any other court communication in their archived files, were included in the analysis.

### Design of analysis: areas of comparison

A content analysis of the FPIs was performed with a focus on the three main areas of case description, gender comparison and time comparison, including six sub-areas in total.

Case exploration:

- aspects of the forensic psychiatric data collection: how and from whom data was gathered in the respective cases;
- how the person was described within the FPI, what biographical areas were focused on, and how the following were described: (a) mental functioning (including intellectual functioning, personality), and (b) everyday functioning (including work, family life, social relationships);
- how the crime and its context were described and explained within the FPI;
- conclusions and recommendations given in the respective FPIs, and subsequent sentences;

Gender and time analysis: similarities and differences:

- are there similarities and differences between the cases from a gender perspective?
- are there similarities and differences between the 1930s and 2020s in FPI procedure and report?

In the gender- and time analyses, there was a specific focus on whether references were made in FPI reports to contemporary scientific framework and to moral values without theoretical or empirical basis.

## Results

### The information gathering within FPIs

The mental states of both M and W at the times of their respective crimes were considered to meet legal requirements for criminal exemption from punishment (i.e. eligible for forensic psychiatric care), and they were sentenced according to the FPI recommendations. The woman, W, was examined by a leading forensic psychiatrist at the time (Professor Olof Kinberg). Her FPI included a long period of observation, psychometric testing and several reference letters or interviews with persons who had known her from child- to adulthood. During his FPI procedure, the man, M, was evaluated by three different psychiatrists. The first psychiatrist was undecided and considered that more clinical observations were necessary; the second concluded that M was not to be considered exempt from punishment^4^ but argued for a reduced sentence according to Penal Code 5 chapt §6.^5^ Nevertheless, after M had spent several months in a hospital, the third psychiatrist (another contemporary Swedish expert, Professor Bror Gadelius) argued for exemption from punishment based on Penal Code 5 chapt §5. Due to lack of witnesses to the actual moments when the shootings took place, it is important to note that all information presented in the FPIs concerning this part of the narrative comes only from W and M themselves.

### Contents of the 1930s FPIs

The FPI reports around the 1930s were not, as today, written according to a standardized format (including required areas under fixed headings). Nevertheless, the two cases in focus were representative of FPIs from the 1930s.^6^ Some issues were almost always brought up, or at least noted as ‘not relevant’ for the case in question. One of these central aspects was to establish the level of intellectual capacity by the structured testing used at the time of the FPI (e.g. using Binet’s scale), which is also investigated historically through references (e.g. grades previous teachers, school-friends, parents). Personality was another central area of investigation, which included patterns of affective functioning, both regarding extreme affective expressions and whether the person displayed too ‘low reactivity’ during an extreme situation. Hence, affective patterns perceived as deviating from what would be considered a normal reaction according to contemporary social standards were of interest. Other central areas always noted, and often described in detail, concerned somatic or medical status, biological heredity of psychiatric illness, and historical and current use of alcohol or drugs. The somatic assessment was extensive (e.g. length, weight, eye colour, body type characterization – often based on Kretschmer’s ideal types), searching for somatic diseases that could affect cognition (e.g. syphilis: Wasserman’s test). Furthermore, in 1930s FPIs, in the borderland between medical or somatic and personality factors, there were often descriptions of the investigated person’s morality being associated with both biological aspects of heredity (e.g. diseases, ‘inferior ancestry’, poor nutrition) and social or environmental factors related to biology and personality. Morality appeared as a separate area of investigation, since it was always touched upon, tested separately, and associated with biological functions as well as personality. For example, a structured, moral investigation was done to ascertain the person’s level of moral maturity (i.e. level of moral maturity reached). Non-structured moral assessments were also made, establishing the person’s level of moral reasoning, which the psychiatrist based on conversations with him or her.

## The content of the FPIs: in-depth case descriptions

### The woman: W

#### Background

W was in her early twenties when she shot her ex-husband with a pistol. She came from a wealthy family and had siblings. No indications of hereditary psychiatric diseases were found within her family, although the mother was described as unemotional and asthenic. W had described her relationship with her mother as strained, contrary to her warm relationship with her father (who had died in a car accident during W’s early teens). She was described as a ‘feeble child’ and quite spoilt. Her school attendance had been fragmented due to her family’s frequent travels abroad (e.g. vacations, business trips). She went to boarding schools with house-keeping or management as a focus, and referees (e.g. her teachers) reported her as having displayed a lack of engagement and low working morale.

#### Cognitive and personality functioning

Based on reference material from family members and acquaintances, W was described as an egocentric and self-involved child or youth. When she was corrected, she did not show indications of shame or remorse, but defiance instead. She was described as a ‘leader’ of a group of girls involved in mischief at school (including a theft) and was characterized by teachers not as untalented but as uninterested in schoolwork and ‘rather aimlessly flitting from old pleasures to new ones’. She was described as emotionally unstable, sensitive to stress with hysterical traits, and in her late teens was admitted to a hospital due to nervous problems. As an adult, she was described as shallow, quite sensationalist, seeking others’ attention and approval, being easily led by others and dependent on them, but also as defiant and stubborn when criticized. Her moral ability was considered underdeveloped and her constitution as asthenic, characterized by unwillingness to work, being easily tired and irresponsible. She had a history of accidents: three car crashes with subsequent hospital care, where at least one seems to have been intentional (driving into a tree when distraught after an argument with her husband). She had been admitted to a psychiatric hospital after a suicide attempt (about one year before the crime). There was no mention of friendships. She met her husband during her mid-teens and was considered to have adopted a dependent attitude to him.

#### Relationship with husband

According to the FPI, her husband had already started courting other women during their luxurious honeymoon (lasting for nine months and entailed high alcohol consumption). When they returned to Sweden, they settled in an apartment. Her husband started a company with W’s finances. She gave birth to a son about a year after the marriage. She did not work, either outside or inside the home (servants kept the apartment and cared for the child), and lived comfortably on an inherited fortune. The matrimonial disharmony escalated after the honeymoon. A continued high alcohol consumption and many quarrels were noted, including verbal and physical violence, intense reunions and threats of suicide from both parties when one of them wanted to leave the relationship. The husband’s gun was repeatedly used in their arguments, where he sometimes threatened to take his life and/or carry out an extended suicide (killing himself and their son).

The husband was described in detail, principally as a charming womanizer, and W craved his attention, but her family opposed their relationship. She married him against her family’s wishes as soon as she reached her majority (about four years before the crime). Based on his actions described in the FPI, he comes across as callous. For example, during their honeymoon, he did not travel with her to Sweden when she needed medical care after a car crash in France. Instead, he stayed there, spending money on women and gambling. Finally, they filed for divorce, a process in which W was aided by her lawyer and physician. But, during the proceedings of this divorce, her husband tried repeatedly (even with threats of violence) to get W to resume their marriage.

#### Crime

The crime occurred in his home where W had been persuaded, under threat of suicide, to visit him. In the week preceding the crime, there had been several verbal arguments and physical violence between them. According to W, the man started to convince her to take him back as soon as she arrived his apartment. At the FPI, she described how she was held there against her will. They consumed ‘quite a lot’ of brandy, and she reported that the man raped her repeatedly. The gun was brought out several times during the time she was there (afternoon to midnight). She described the events leading up to the shot: an argument when the man held the pistol, aiming it towards his chest, urging her to kill him if she did not resume their marriage. She said that suddenly she held the pistol and pulled the trigger ‘mechanically’ and ‘without will’, obeying her ex-husband’s voice. W described her state of mind at the time as confused due to fatigue, alcohol, threats and rapes. Thereafter, she called the police (00.10 a.m.), her lawyer and her physician stating that she had shot her husband. When the physician and police arrived, they described her as ‘very hysteric’, distraught and somewhat incoherently repeating ‘I shot him, I shot him’, and ‘I shot him because he wanted to rape me’. At approximately 02.00 a.m., the hospital announced that the husband had died.

#### The FPI: mental functioning

W’s cognitive ability was tested, with results within the normal range, but with a low level of general knowledge. She was deemed as unpractical, with low persistence. Regarding mental health and personality, she was described as constitutionally weak, passive, amoral or immature or inconsiderate, pleasure-seeking and hysterical. The forensic psychiatrist viewed the case as a combination of unhappy environmental (including situational) influences and a constitutional weakness, summarized as follows:

It is certain that the unhappy development of these two persons’ fates show that the life of the rich, if it is wasted in idleness and without fulfilment of duty, if it is ruled by pleasure-seeking and abuse of poisons, if it is led in such a way that almost all rules for a healthy life, both bodily and mentally are violated, if it lacks sensible goals, interests outside the individual and a sense of commonality with the whole, easily derails into a senseless ‘witch-dance’, where the sense of what humans with a maintained decency may allow themselves, after a while is eradicated completely and where the human individual is exposed to be a victim for almost any kind of delusions.

The conclusions of the FPI were that W had committed the crime during a ‘state of psychological weakness and illness that, regarding its effects on [her] actions, was to be considered equal to mental illness’ (in the sense of ‘legal insanity’). The forensic psychiatrist also recommended that W should receive treatment that, according to common practice, was allotted to such accused persons (i.e. she needed mental hospital care). In the 1930s in Sweden, psychiatric diagnosis was not made according to a standardized nomenclature. Instead, a personalized diagnostic description was given, and W was diagnosed as ‘a constitutionally abnormal, hysterical, psychoneurotic individual, who several times has suffered from mental illness’.

### The man: M

#### Background

M was 37 years old when he shot his wife with a rifle. His father had a university exam (undisclosed type), was a well-respected teacher, and ‘known to be very gifted and of great willpower’. His mother was considered intellectually feeble (‘imbecile’), religiously brooding, suffering from periodic depression with possible manic episodes, and she had committed suicide when M was a child. Three close relatives had long-term stays at a psychiatric hospital, with one presumably committing suicide (unclear intent, sleeping pill overdose). M also had a sibling who had ‘done well in his life’.^7^ His relationship with his parents and family was not further described in his FPI.

#### Cognitive and personality functioning

M had difficulties in school and had to repeat one year since he did not meet the course year requirements. Nevertheless, he managed to complete a degree in agriculture (MSc), but with ‘great difficulty’. In testimonies given by other students on the course he was considered ‘not gifted but very diligent’, while his sibling described him as lazy. After graduating, he worked on an estate where his uncle was in charge; he left after some years to manage another estate. Regarding childhood personality, M was described as ‘feeble, soft and compliant, reserved, volatile and fickle’. During his higher education, course comrades described him as ‘extremely suggestible, quiet and withdrawn’, ‘kind and submissive’, but he could also be very stubborn and sometimes difficult to ‘come to terms with’. According to a relative, he ‘often exaggerated difficulties regardless of how small they were’, and ‘sometimes it was as if his train of thought just “stopped” and he rehashed the same thing over and over again, especially when he had been drinking’. Overall, his personality and behaviour changed when he drank alcohol. When intoxicated, he had on several occasions behaved in a socially strange way and ‘acted as though he was not completely normal’.^8^ He was described as drinking more frequently during his marriage, but rarely at home.

#### Relationship with his wife

At 30 years old, M married. His wife, then in her early twenties, came from a wealthy family. The couple moved to his mother-in-law’s estate and, throughout their relationship, his wife paid for all expenses. The marriage was described in the FPI as initially good, but then turning bad due to M’s ‘periods of dark mood’ during which he often talked of suicide and continually sought comfort from his wife. He was afraid to sleep alone and needed her presence (or one of the children) in bed for comfort. His insecurity and obsession with suicide was described as highly irritating for his wife, causing arguments. A few years into their marriage, she started to spend more time with a family friend, Mr X. The wife and Mr X were described as getting closer over the years and, while she was alone on vacation with one of their children, Mr X visited her overnight. After this, M and his wife spent more time separately but did not divorce. In the FPI, the wife was described by M’s brother as ‘snappy’ towards M, which the brother considered to be ‘her usual way’.

#### Crime

M shot his wife one night at the couple’s home. They had lived apart for a while, and she was coming home for one night before leaving to stay with Mr X. That day, while M was hunting with friends, he had been observed to ‘grimace, sitting on a stump and twisting his hands around his rifle’. M later said that at the time he considered suicide, and he continued to do so for the rest of that evening, including preparing for suicide (e.g. sorting his important papers). During the evening, his wife packed her things, and they argued about Mr X. M was very stressed and he smoked cigarettes ‘constantly’ throughout the day. He had drunk 10–15 cups of coffee and a considerable amount of alcohol. His anxiety escalated when his wife did not go to bed with him. M went back to her repeatedly, asking the same question (i.e. if she would come to bed soon), which finally made them both become very irritated. A witness, the children’s nanny, said that he suddenly fetched his rifle and stood still, aiming at his wife. His wife did not appear afraid, but then M shot her in the chest. She died in the hospital about one week later, the bullet having caused a severe infection. At the hospital, she said that she had gone into her husband’s room to fetch some of Mr X’s books, where M had grabbed the rifle. After an extended process of M loading the rifle in front of his wife, he said ‘You do value your life don’t you?’, and aimed his rifle at her for a long time. She said she did not think that M would dare to fire and reported that ‘he had threatened her before in a similar manner, but had never dared to do anything when she had looked at him’. The witness had heard the wife yell ‘Miss!’ (i.e. calling the witness) and described seeing M aim the rifle at his wife who then lay wounded on the bed, whereupon M fell over her and repeatedly screamed ‘I’m sorry!’. He was perceived as shaken and acting in a confused manner. The wife was transported to the hospital, and M travelled to his father after a few days. The family agreed to keep the crime a secret if the wife survived.

#### The FPI: mental functioning

M was assessed by three psychiatrists: the first while in prison, and the second in a psychiatric hospital after a recommendation from the first. The third expert was consulted since the previous two had come to different conclusions. During the first assessment, all the main information usually included in the FPI was collected, but, according to the psychiatrist, no conclusion was considered possible without a period of observation at a psychiatric hospital. The second assessor, the hospital psychiatrist, added his own impressions of M, but did not add information from new sources regarding M’s background. The third expert added his own impressions and also added information from one referent (M’s sibling). M was considered to have heredity for both cognitive deficiency and affective disorder of a principally depressive nature with anxiety and lapses of impulse control or suicidality. He was considered to somatize his psychiatric problems, which the FPI noted as complaints about numerous ailments (e.g. ‘always pressure around his head’) related to anxiety. M claimed to have had suicidal thoughts, escalating in intensity, during the six months preceding the crime. He did not have any suicidal thoughts while in custody, but he was certain that they would reappear when he was released. Regarding intelligence, M was described as relatively normal, but no intelligence testing was conducted. Instead, the main focus of the FPI was on his personality, emphasizing that M was terrified of being alone and got particularly anxious when about to go to sleep. In the expert assessment, the sibling described the family as more worried for M when he did not complain, that he had become increasingly apathetic during the approximately six-month period preceding the crime; the family had then feared suicide. The three psychiatrists who assessed M came to the following conclusions.

(1) Prison psychiatrist: M was considered to externalize responsibility, not showing any remorse and feeling sorry for himself. M maintained that it was an ‘accident that he could not have prevented’. One thing described in much detail was M’s preoccupation with a book presenting ideas that, according to M, explained the crime: that not having sex creates diseased states in men. Thus, M concluded that ‘his wife’s coldness and refusal of “eroticism” had made him completely unhinged’. However, the psychiatrist considered observation to be necessary, recommending M to be sent to a psychiatric hospital for further assessment.
(2) Hospital psychiatrist: The second psychiatrist (at the hospital, after observation) considered that the requirements for legal insanity (according to Penal Code 5 chapt 5§) could not be applicable to an ‘explosive act of emotion committed while under such a psychological state of tension’. He considered that, instead, M must have been in such a state of mental abnormality as described in 5 chapt §6, as to be entitled to some kind of leniency due to extraordinary circumstances.
(3) Expert psychiatrist: The third psychiatrist, Professor Bror Gadelius, described how M blamed his wife for him having committed the crime due to sexual frustration, but Gadelius remarked that he considered M’s ‘sexual need never to have been particularly strong’. One hypothesis advocated by Gadelius was instead that M’s proclivity to read sensational stories, often love dramas that ‘were always on the newspaper’s front page’, could have affected him and boosted his strong reactions. Gadelius concluded his investigation of M as follows:

M is from a psychological perspective a disharmonious developed personality . . . . He is one of the ‘feeble minded’ [Swedish: *debil*] badly equipped for life and its stressors. His urge to commit suicide which M talks about so much is in fact in his blood and probably also the rashness and impulsivity that caused his plans to commit suicide to derail and be directed towards his wife. The sexual problems, about which he himself talked so much, could in a certain manner be related to his ‘worried and “fumbling being” and the un-manliness within him that has caused his wife to lose her affection for him’.

Gadelius does not consider M’s reactions within the current living situation as behaviour a ‘sound man’ should display:

The manner in which he reacts before the wife’s averseness and her obvious approval for another man was not normal for a sound man, because, instead of directing his anger towards Mr X, it was directed in an almost ridiculous antipathy towards Mr X’s books.

## Analysis of case similarities and differences: gender

Information for M and W was gathered from similar numbers and types of sources, including several referees, both those in close relationships and those who had superficial acquaintances (current and historical). No considerable differences were found regarding the depth of analysis, other than M being assessed by three psychiatrists while W was assessed by a leading expert from the start. This multi-source information for M and W was combined into a ‘life-narrative’ for each of them: behaviour during the crime and the perceived mechanisms behind it were related to the developmental history of their personality and character maturation, as well as the psychosocial context of the crime – all were integrated into the psychiatrist’s narrative presented in the FPI report.

### The person, the situation, and their interaction

Long-term tensions, with infidelity, jealousy and violence, were present in both relationships. At the time of the crime, the situations had come to a head and both couples faced the choice of either continuing together or separating for good. Both M and W shot their partners during arguments concerning their relationships, where one of the partners felt a strong desperation or stress before the prospect of being left by the other. The difference was that in M’s case the perpetrator was the desperate one, while in W’s case it was the victim. Both M and W had consumed a considerable amount of alcohol and reacted in a similar manner after the shooting: being confused and distraught, with no intention of fleeing or concealing the crime. Both W and M externalized guilt and blamed all their misery, and the reason for the shooting itself, on the victim. Both were also considered to be sexually sub-normal, and sexuality and impending break-ups were noted in the FPI reports. Sexuality was commented on explicitly as an important causative factor behind the crime, but the offenders in each case were in opposite ‘stereotypical roles’ of such an argument: W’s husband was pleading with her to resume their marriage, while M was soon to be left by his wife. Therefore, in M’s case, he considered his wife’s refusal of sex had driven him to the crime. W, on the other hand, stated that her ex-husband raped her twice that day and that these rapes were part of the cause of her confusion that led to her shooting him, and that she ‘shot him as he was about to rape her’. Despite these differences, prominent personality traits of submission and dependency towards their stronger (at least as presented) partners were characteristic of the offenders; both victims were portrayed as the dominant person in their respective marriages.

### Personality traits

Both were diagnosed with personality-related problems but neither was considered psychotic. An in-depth description of their characters was presented (including changes through life), and dominant personality traits for both were being vague, shallow, seeking others’ approval, interpersonally dependent and easily influenced by others, and experiencing anxiety. Both were also described as having somatizing mental reactions, and they had a history of treatment episodes for various (psycho-)somatic disorders. They had previously been treated for affective disorders as inpatients at a psychiatric hospital and repeatedly proved to be suicidal. They were described as exaggerating their misery and others’ animosity towards them. There were also some character differences: W was portrayed as considerably more outgoing and alternated between anxiety and aggressive acts towards others, while M was described as ‘weak’, sad, often thinking about suicide, and seeking comfort from his wife (despite his aggressive outbursts during intoxication). These patterns were mirrored in their previous hospitalizations, when M had shown considerably more internalizing patterns such as worrying and depression (including stunted aggressiveness), while W displayed patterns similar to today’s borderline personality syndrome, that is, externalizing, being emotionally unstable and having what, in the 1930s, were termed hysterical traits (e.g. partial paralysis, screaming fits). Both had problems with being contradicted but they reacted differently, with M showing sadness and defeat (while not intoxicated), while W became defiant and desperate. Both were considered to have had an improper and/or unhealthy use of alcohol, and when intoxicated they behaved in a very headstrong, impulsive and self-destructive manner.

### Cognitive functions

Both M and W had psychologically unstable mothers and successful and intelligent fathers, but in M’s case her mother was also considered intellectually ‘deficient’. They were both described as psychologically and emotionally unstable and weak as children (especially M). W underwent an intelligence test as part of her FPI, which M did not. This is interesting, since it was M, not W, who had shown learning difficulties as a child in school and as an older student at university. Based on hereditary theories of intelligence published in books on psychiatric disorders by experts in both M’s and W’s cases (Gadelius, 1924; Kinberg, 1930), M should have been tested due to a higher hereditary risk (i.e. his mother) for cognitive problems.

### Self-support

Both M and W came from privileged backgrounds, which was (and still is) relatively unusual among individuals subjected to FPIs. W was economically independent, and her husband and victim benefited from her wealth, in contrast to M. He had to work for his living on his wife’s family’s country estate as a salaried agronomist, and his wife was rich. Hence, both relationships were economically unequal, with the women economically independent, meaning that at least in theory they could dictate the economic terms for the couple.

### Description of the victim and victim or offender relationship

Both victims were reported to have ‘provoked’ M and W, urging or daring them to shoot. Both victims had had extramarital affairs and were portrayed as having played an active part in escalating the course of the argument. Apart from this, the victims were described in different ways. W’s husband was described in a manner that made him appear as a scoundrel and womanizer (e.g. living on her assets, exerting strong power over her to better his own life, without true concern for her health). In M’s FPI report, there are descriptions of his wife’s irritation and antipathy towards M when he was seeking assurance and confirmation. The wife is portrayed as harsh and unfaithful, as aloof and frustrated, wanting to end a relationship with a dependent, sad husband whom she did not consider as ‘a real man’. Both relationships followed a similar pattern with happy starts, but after some time the couples had recurring quarrels which, in the end, included physical violence and the presence of guns (although never fired until the final quarrel). Although M’s and W’s standpoints in their relationships with the victims may seem opposite – M wanted to resume his marriage, while W was seemingly pestered by her ex-husband to get back together – the question of resuming the relationship versus separation was the crucial issue when both weapons were fired.

### Values and gender-related information within FPI conclusions

Value-oriented comments occurred often in these two FPI reports, including in diagnoses and legal conclusions, and these were sometimes moralizing, indicating the psychiatrist’s perceptions of markers for ‘a healthy’ man and woman. M was described as ‘not a sound man’ due to not having handled the situation in his ‘vile’ marriage the way a sound man should have; this was described as to ‘attack [*sic*] Mr X physically instead of [as M had done] endure a continued contact with him and engage [his wife] in an argument regarding Mr X’s books’. W was described as having a ‘very immoral way of life’, ‘drinking much alcohol’ and ‘other unsound habits for body and soul’. In W’s case, it was not clear whether her habits were unsound for a woman specifically, but definitely considered as ‘unsound’ for a person in her social position. In the psychiatrist’s formulations, it was rather her violation of Victorian norms (including social class) through lethargy, disinhibition and the ‘duty of keeping healthy’ (see Johannisson, 1990: 42–64) than W being a woman.^9^ Nevertheless, there were gender-related issues regarding expressions of psychological distress in the FPIs, and features in M’s and W’s psychiatric disorders were considered by the experts to be an anathema to contemporary gender norms. The descriptions of M by his wife – a ‘disappointment’ and ‘not a real man’, which she tied to his weakness, worry and constant need for attention from her – were considered as evidence confirming his unmanly traits. W was described by referees from her childhood as a leader of a group of girls who, in search of excitement, once stole a wallet from a teacher. W’s role in this was seen as opposing contemporary female norms of submission, self-control and gentleness (e.g. Johannisson, 2015). From historical analyses of women from the higher socio-economic groups of society who were treated in Swedish psychiatric hospitals during the 1930s, certain acts of rule-breaking (e.g. masturbation) were deemed more relevant indications of mental instability for women than for men. Johannisson (2015) concludes her in-depth case analysis of three women from higher SES groups treated in mental hospitals in Sweden that the range of normal behaviour was narrower for women than men at the beginning of the twentieth century, and especially so regarding expressed sexuality, but also particularly concerning eccentricity, expressing grandiose and arrogant behaviour, or acting out (e.g. screaming at others, being rude, losing self-control). Also, regarding alcohol consumption, what the assessing psychiatrists considered socially or morally wrong was not the amount of alcohol consumed, but principally the type of intoxicated behaviour. When M became drunk in restaurants or at family parties, he disturbed others and behaved inappropriately (e.g. nagging, boisterous). In the FPIs from this period, open display of impulsive behaviour while intoxicated was a core aspect of handling alcohol ‘in the wrong way’ (especially for higher SES groups). Previous historical research (Hildebrand Karlén, 2022; Johannisson, 2015) shows that this was a view present in more than the cases of M and W. Thus, it is possible that when W engaged in alcohol consumption that was similar to M’s, the psychiatrist’s association to her having a morally reprehensible lifestyle in general might have been due to W being a woman whose behaviour was measured against different behavioural norms than for M. Such a division between ‘not mentioned’ for men and ‘mentioned as morally wrong and/or pathological’ for women regarding certain factors has been noted in previous research on contemporary journals from psychiatric hospitals in Sweden. An example from Johannisson’s investigation is that a man’s frequently occurring masturbation was only stated in his journal as present and was discussed with the doctor as a way of solving problems concerning sexuality, while for a woman masturbation in itself was considered a sign of pathological homosexuality or bisexuality; according to Emil Kraepelin, it was a threatening sign of a slide for a woman towards male sexuality (Johannisson, 2015: 230).

Another interesting aspect in the cases of W and M, with relevance for the notion of gender norms, is that it was the women who were economically independent, which was rather unusual at this time in Sweden. W and M’s wife had the economic possibility, unlike many other women at the time, of separating from their husbands. How this could have affected the self-image of the men in these particular cases is not clear, but it is plausible that it affected them negatively, given that the traditional gender roles of the time in question were reversed in their marriages. Both husbands in these cases were about to lose not only their wives but also their economic security, and in M’s case his house and work (as agent of their estate); this situation certainly impacted the relationship dynamic, but perhaps especially so in M’s case due to his high anxiety baseline and sensitivity to change.

### Summary: gender and FPI praxis around 1930

In line with praxis in most FPIs around the 1930s, no standardized diagnoses were made for M and W. From the personalized diagnostic judgements presented, both were considered to suffer from personality-related problems that had caused them, under these highly stressful circumstances, to act out violently. Some differences regarding diagnostics between them can also be seen in the FPIs reflecting notions more about gender roles and social position then about diagnostic categories. For example, W’s problems were considered as an expression of a hysteroid disposition, and judged as unfit to meet life’s demands due to having grown up spoilt in a wealthy family where she had not taken any responsibility for her life. The combination of a propensity to react in hysterical ways and inability to take responsibility left W badly equipped to handle life’s challenges. M, on the other hand, was described as lacking virility, being prone to rumination, being depressive, dependent, and unable to take charge as was expected of a man in his position. Thus, the diagnostics are heavily related to aspects of how M and W deviated from what would have been expected of them regarding their gender roles and social positions. That W was assessed by an expert immediately, while M was assessed by two other psychiatrists before meeting an expert, also generated some case differences. Although both received forensic psychiatric care according to Penal Code 5 Chap §5 (i.e. legal unaccountability), the second psychiatrist instead considered leniency and not insanity in M’s case, indicating less clarity in how to consider his perceived pathology in legal terms. The reason for this discrepancy between these cases was unclear from the arguments in their FPIs. It could, of course, be due to the fact that these cases were difficult to assess from a legal standpoint, but nevertheless it does not alleviate suspicions that it was regarded as more unexpected and pathological of a (wealthy) woman to kill her partner than for a rejected man to do so.

## Analysis of similarities and differences: time

The most striking overall difference in FPI report focus over time is that the presentation of a detailed narrative in the 1930s, resembling a full biography including information from several referees, changed into a focus on the individual’s current psychological symptoms and functioning. Hence, the description of the crime, the person and his or her life history, and the mechanisms regarding how the person came to commit the crime are considerably less detailed today, but are more clearly referenced in empirically founded documented information (e.g. psychiatric care journals, social services contacts, etc.). One reason for this change from a comprehensive narrative to a much more focused report is probably that one psychiatrist is no longer solely in charge of the FPI. Today, a psychiatric team of experts (three to four professions represented) works from their respective professional perspective to outline parts of the person’s life relevant to the current crime. Each expert then writes their own report. The team structure with multiple reports presumably hinders the assessment from becoming *one* narrative, especially since different professions may consider different theoretical aspects to be relevant for understanding the person and their crime. However, an agreed narrative could arise since the team works together during the course of the FPI, discussing their progress, obtaining results and evolving hypotheses. The characterization of the person ‘as a whole’ may therefore emerge as less clear today, and in all probability also less sensitive to *one* psychiatrist’s individual ideas (including stereotypes and bias) than in the 1930s. Another overall change over time is that information is today presented with less interpretation by the psychiatrist (i.e. keeping conclusions closer to empirical findings) compared with what is seen in the FPIs of the 1930s, when personally held values could be included more freely to support conclusions. It was clear that the included value judgements in these cases influenced conclusions^10^ (e.g. lack of more profound character; only engages in shallow diversions/‘nerve sensations’);^11^ such moral condemnations by the psychiatrist of the offender’s or victim’s behaviour, character or lifestyle would not be tolerated today, according to current FPI report guidelines (HSLF-FS 2015:31) (see Note 3). Naturally, this does not mean that today’s FPIs are devoid of value-laden words or reasonings, but it would be difficult nowadays for a Swedish FPI expert to get a report containing clearly moral-/value-oriented statements (as exemplified in M’s and W’s cases) without an empirical basis accepted by colleagues and the court. Of course, it should be noted that some terms that are used in today’s FPIs will, in the future, be considered value-laden and as expressions of ‘immorality’, and some assumptions and measures will be considered as not ‘empirically based’ any more. For example, to use the word ‘immature’, which can sometimes be seen in FPIs from our decade, is a form of, if not moralizing, at least a value-laden word since it is the negation of a norm that is considered as positive. Also, the term ‘immature’ lacks a clear definition that separates it from ‘mature’. Instead, as Sadler (2005: 213–16) suggests, description of patterns that the judgement is based upon could replace the use of vague terms with negative moral connotations within diagnostic reasoning. Descriptions to replace ‘immature’, depending on what aspect is accurate for the person in question, could be: has problems interacting with persons his or her own age; seeks the company of persons about 15 years younger; feels himself having most in common with persons 15 years younger than himself.

### Changes in information gathering and reliance within FPIs

In both the historical cases, the psychiatrist collected a large amount of information from a broad array of areas, and contacted several referees. Today, a similar broad base of information sources is used, such as clinical observations, interviews with the offender, standardized testing, self-report forms (results compared to norms), but only rarely interviews with referees. Information sources considered of particular importance today are: meeting and interviewing the offender, and observations in the psychiatric ward where the FPI is conducted (Svensson et al., 2022). The main differences in information gathering between 1930s and 2020s is how referees are contacted and which information is considered relevant to the FPI. Regarding the referees contacted in the 1930s, some referees had a close relationship with the offender (e.g. family members), but others only knew them many years ago (e.g. teachers), and other persons who may – or may not – know anything at all about them (e.g. neighbours) were also approached. The referees were sent a letter and completed a written, standardized form^12^ that included several areas of the offender’s behaviour and psychological or social functioning. The requested information was partly of an empirically verifiable nature, but also included personal judgements of the assessed person’s behaviour and morality. To ask referees such questions today is considered unethical and does not hold explanatory value within an FPI. Today, oral interviews with select referees are made in some cases but the requested information is then specific to the case and to the SMD assessment. To send out forms indiscriminately to potential referees would today constitute a breach of secrecy since, by doing this, each recipient is informed that the offender in question is undergoing an FPI.

### Changes in content: specific areas

In the FPIs of the 2020s, the emphasis is on psychiatric symptomatology, on cognitive and personality functioning, and on general lifestyle and everyday functioning. All these areas were noted in the 1930s FPIs, but now the focus in these areas is mainly on *the time of the crime and during the FPI*, not comprehensively describing the person’s history in these areas and development up to (and including) the FPI. This results in a considerably shorter and more standardized FPI report in the 2020s, aided by it containing sections under specific headings. Despite similarities, some areas emerged as main examples of changes in FPI content over time.

### Sexuality as an explanation

Problems concerning sexuality were cited for both W and M, but it was not considered relevant to their crime as a ‘biological’ explanation by either psychiatrist.^13^ However, in the 1930s their sub-functional sexuality seemed to be considered relevant to psychopathological processes, and as an explanatory model argued from a somatic stress and tension perspective as well as a more diffuse idea of mutuality.^14^ Today, sexuality from a biological ‘stress/tensional’ perspective is not used as a possible explanatory model for violent outbursts (especially not from a biological stress or tension perspective). However, problems with social interpretation, such as when misinterpreting interaction as a sexual invitation, could be included within a broader ‘social interaction problem’ in today’s FPIs.

### Personality and intelligence

Long-term personality dysfunction, including affective lability, was emphasized in both M’s and W’s FPIs.^15^ Personality and cognitive ability were assessed by the psychiatrist in the 1930s, but are now assessed by the forensic psychologist. While results of cognitive testing related to norm group data figured in FPIs of both the 1930s and the 2020s, personality assessment was less standardized in the 1930s. Today, personality assessment often includes use of standardized measurements to highlight personality traits from a typological and dimensional perspective, including a norm group comparison. In the 1930s, personality assessment was more clearly blurred with social values and moral opinions, and no structured forms or comparisons with norm groups were used. Today, cognitive testing is often done, and considered necessary if there are indications that a low intellectual functioning could have affected the offender’s behaviour. A structured intelligence testing was almost always made around the 1930s in Sweden, but in our two cases only W was tested. Since it was M who had not met course demands and struggled in school, he would today most likely have been tested. M’s cognitive ability would probably also be considered more explicitly today when discussing his affective instability and impulsivity, presumably since there is a significantly larger theoretical focus within psychiatry today on children’s neuropsychiatric development and how they relate to cognition later in life.

### Alcohol intoxication

Both M and W were alcohol intoxicated at their crime, while M also had over-consumed stimulants (i.e. nicotine, caffeine). In both FPIs, descriptions of earlier alcohol habits were presented and, in M’s case, one of the psychiatrists even tried to estimate the exact amount consumed, which indicates that the impact of the alcohol was considered relevant. To assess whether the offender has been alcohol intoxicated and decide on its potential impact on functioning at the time of the crime is still important. Today, somatic tests are also conducted to investigate drug habits (e.g. urine analysis, hair analysis), while fewer options were available in the 1930s (e.g. tests of liver functioning). Neither M or W was diagnosed with *Alcoholismus chronicus* (as described by Huss, 1849), even though there are several indications in their FPIs of an alcohol-use syndrome according to current diagnostic standards (‘Substance use disorder’, American Psychiatric Association [APA], 2013), mirroring a lower diagnostic threshold in this area today (see Hildebrand Karlén, 2022).

### Somatization and the decreased focus on the body

Generally, the body was in focus to a considerably larger extent in 1930s FPIs, as a focus for both description and assessment (e.g. several more tests and measurements), but also to underpin the psychiatrist’s hypotheses regarding types of psychological dysfunction and suffering. Thus, it appears that the border between bodily and mental symptoms was less distinct in the 1930s. For the majority of cases in 2020s FPIs, somatic description and examination, as well as a discourse of somatic causes for psychological manifestations, are virtually non-existent, and somatic tests are only made on clear indications – not routinely. This can be related to the decreased explanatory use of biological models for psychological symptoms, presumably partly influenced by decreased importance (e.g. less spread of viruses today that can affect psychological functioning, seen in less testing for syphilis) and partly due to the perceived less-clear explanatory value regarding how somatic tension can have a psychological impact relevant to impulsive and violent acts. As a consequence, the importance of assessing the body, besides testing for tumours or genetic disorders creating severe hormonal disturbances, is considerably less within psychiatric explanatory models in the 2020s. In contrast, in the 1930s the somatic examination was highly structured (height, weight, eye colour, skin condition, etc.), including a somatic ailment anamnesis and, importantly, also describing the shape of the body. This focus was based on perceived explanatory ‘empirically based’ scientific models at the time, such as Ernst Kretschmer’s model, which was popular in Sweden.^16^ This is an example of how explanatory models – perceived as scientific at the time of creation but discarded by later research as based on faulty assumptions – shape the content and focus of psychiatric assessment.

### Writing about persons close to the offender

The behaviour and lifestyle of the offenders’ partners, who were the victims, were described in detail in these FPIs. Today, the in-depth level of description, as well as the value judgements seen especially in W’s case, have been abandoned, and the emphasis is nowadays on the assessed person. The focus on personal integrity, also reflected in today’s guidelines for FPIs, have presumably contributed to this development. In comparison, regarding children, it was recorded that both M and W had children, but parenting ability was not noted or evaluated in either case; presumably this would be an important factor to consider in the social worker’s investigation in the FPIs of the 2020s. M and W both displayed inappropriate behaviour towards their children, which today would have resulted in a report to social services, but at the time of the 1930s FPIs was not discussed.

### Diagnosis and conclusions reported to the court

The diagnoses in the 1930s were formulated as descriptions of the person’s perceived problems and/or complemented by using dimensions of various personality traits from contemporary theories. This differs from today’s use of a standardized diagnostic nomenclature (in Sweden: the *Diagnostic and Statistical Manual of Mental Disorders* [DSM] system), where symptoms and specific behaviours are the focus, making the characterization of symptomatology clearer but missing ‘the whole’ – as reflected in the 1930s narratives. Instead of finding the ‘right’ diagnostic category (see e.g. the discussion by Frances, 2013), a focus on the dynamic interaction between the many specific within-individual risk factors for psychiatric distress may illustrate what the synergies between these risk factors mean for the person’s ability to handle specific high-stress situations and manage everyday life. The importance of considering such interactions for more accurate clinical decision-making is obvious, based on research that has led up to risk-assessment instruments for violence (e.g. the Historical Clinical Risk Management-20, Version 3 [HCR-20V3]; see Douglas et al., 2014) as well as to investigate the level of societal support an individual needs (the Level of Service Inventory [LSI]; see Andrews and Bonta, 1995). An important drawback of basing psychiatric assessments and diagnostic conclusions on overt symptoms and behaviours (which has dominated since the DSM-III’s diagnostic re-conceptualization in the 1980s), also needs to be considered here. Since the DSM-III in the 1980s, reliability has been consequently and increasingly, emphasized over validity (see Frances, 2013; Hildebrand Karlén, 2013). The focus on symptoms in the form of delimited overt or observable behaviour has made psychiatric assessments more ‘superficial’. Due to the need for ‘observability’, and to the limited forms of human behavioural expressions for psychological distress, certain psychiatric symptoms are present in several different diagnoses. This results in a certain overlap between diagnostic categories. This argument has contributed to a movement towards a personalized psychiatry and person-focused medicine seen today. To interpret symptoms in light of the ‘whole person’ (including history) is an objective that has been revived today, in order for diagnoses to be more useful to planning and adapting treatment interventions for a person (see Bornstein, 2017; Bram and Peebles, 2014). In summary, psychiatric assessment nowadays is better described as ‘pluralism without synthesis’, while in the 1930s, a more eclectic use of the perspectives of the biopsychosocial model (BPS model) in combination with morality assessment was possible (i.e. a more merged and eclectic use of a biopsychosocial+morality model).

## Limitations and considerations

In this matched-case gender study, structural differences were found that were not necessarily related to gender or social class. M was assessed while in prison and in a psychiatric hospital, but W was only assessed in a psychiatric hospital; she was subjected to several tests while he was not. From the start, she was assessed by one of the leading forensic psychiatrists in Sweden, while he was assessed by two psychiatrists and then the expert. Her case was highly publicized, but it is not known whether his was. It is not possible to decide whether these differences are related to gender, social factors or chance, which limits the possibility of interpretation. Not all aspects that might be considered in today’s FPIs could be compared with the 1930s. Nevertheless, based on the structured headings in the guidelines for FPI reports in the 2020s, the main aspects that they regard as necessary were present in the two 1930s FPIs investigated.

## Conclusions

The development of an individual’s character, and the interaction between character and his or her environment, were emphasized in the FPIs from the 1930s – based on structured assessment methods as well as explanatory models from a biological, psychological, social and also moral perspective. In the 2020s, the BPS model is still reflected in the FPI, perhaps even more so through the organization of FPIs by a multi-professional team consisting of a psychiatrist, psychologist and social worker. There are several reasons for the more selective FPI assessment method and report today; one often mentioned is to protect the integrity of the assessed persons. However, especially in complex cases such as those of M and W from the 1930s, it is often crucial to consider the interplay between different perspectives, such as psychotic-like states induced by an extreme situation preceded by long-term stress. To ignore an in-depth examination of these perspectives can result in a limited understanding of the case, with the court not being presented with the ‘entire picture’. For example, the offender has not performed a violent act in a social vacuum, and the victim’s behaviour may carry important information as to why the offender committed the crime. But it is a fine line between presenting this in sufficient detail (so that the offender’s behaviour can be assessed accurately) and victim blaming. Constant monitoring of FPI reports is necessary to maintain such a balance. Also, in the two 1930s FPI reports analysed here, clear moral condemnations were included regarding lifestyles and behaviour based on contemporary social norms. Moral judgements, including those on gender and social class, were often underpinned by references to vague psycho-biological models in the studied FPIs. Such formulations would not be accepted in the 2020s. The concept of an SMD is narrower and is clearly defined in legal texts today, which should make decisions (at least a considerable bit) harder to base solely on individually held moral and value judgements than in the 1930s. Also, due to the team structure, FPIs today are less dependent on one expert psychiatrist’s notion of ‘normality’ and ‘good behaviour’. However, in the 2020s, the psychiatric field risks compartmentalizing information according to an almost Linnaean taxonomy of overt signs, which carries other problems. By focusing on symptoms and overt behaviours in psychiatric diagnostics underlying FPIs, the complex and often contradictory ‘whole’ image may be lost in favour of factors or lines of arguments that are easy for non-professionals to understand, due to their high face validity. More qualitative research and more historical research could illustrate the consequences of such superficial praxis and compare it to a more holistic perspective, capturing the synergies of thoughts and feelings that drive behaviour (and vice versa). These synergies are an important aspect for professionals working with FPIs – as well as for the courts – to consider to be able make as accurate decisions as possible.

## Notes

1. Swedish: ‘*från sina sinnes bruk*’, literal translation: ‘parted from use of one’s senses’.

2. Swedish: ‘*själsverksamhet*’, literal translation: ‘activities of the soul’.

3. HSLF-FS 2015:31. Gemensamma författningssamlingen avseende hälso- och sjukvård, socialtjänst, läkemedel, folkhälsa m.m.: Rättsmedicinalverkets föreskrifter om rättspsykiatrisk undersökning [The common constitution concerning healthcare, social services, medicine, public health etc.: The national board of forensic medicine’s regulations concerning forensic psychiatric investigation.] ISSN 2002-1054; accessed (14 Dec. 2022) at: https://www.socialstyrelsen.se/globalassets/sharepoint-dokument/artikelkatalog/foreskrifter-och-allmanna-rad/2015-12-31.pdf

4. That is, not suffering from a sufficiently severe type, or degree, of mental disorder.

5. Based on M’s extreme situation at the time and his then desperate state of mind.

6. Based on the first author’s experience during extensive reading of FPIs from this period within the present research project.

7. M’s sibling became a physician who contributed with information that is comprehensively cited in the final expert assessment within M’s FPI procedure.

8. Examples of his strange behaviour during intoxication were: he had threatened to drown himself; and ‘heaped’ his wife/others with verbal abuse.

9. Regarding moral values and social class, Olof Kinberg gives a scathing description of the decadent behaviour of the ‘upper classes’, which can be related to his own, upper-/middle-class standing in Swedish society where industriousness and health were valued. Previous research has shown how the classic Victorian moral values, often held by Swedish physicians and psychiatrists at the time, seeped into physicians’ judgements of what should constitute normality (Johannisson, 1990; Qvarsell, 1982; also, for international aspects, see: Birch, 2008; Tomes, 1994).

10. This extends into a form of ‘cultural criticism’ where a way of life among a subgroup in upper social class is judged harshly.

11. Note that, in the present study, temporal changes in psychiatric terminology, such as describing a certain standardized intelligence level as ‘imbecile’ or ‘idiot’, were not considered as an example of morally subjective judgement by psychiatrists in the 1930s.

12. There were several such referee forms, adapted to family members, manager at work, etc.

13. M was certain that frustrated sexuality was a biological cause for why he had become so ‘beside himself’ and had shot his wife, while Gadelius rejected this explanation, not based on its irrelevance as a theory but based on the fact that he considered M to have ‘weaker than normal sexuality’. Kinberg considered W to have ‘an incapacity for mutual sexual engagement’.

14. However, not from a psychoanalytic perspective. Also, how Kinberg could know this or had investigated this in W’s case is not clear.

15. This general observation was based on descriptions in the FPI regarding W’s sensation seeking and more externalized anxiety with both self- and other-harming behaviours (e.g. suicidality, biting a nurse while hospitalized). M was described as displaying a more depressive, self-directed anxiety, including brooding and suicidal thoughts, inhibited aggression and passivity.

16. This model was also mentioned by both experts Gadelius and Kinberg in their other medical publications.
